# Development and assessment of a nutrition literacy scale for patients with end-stage kidney disease undergoing dialysis and its correlation with quality of life

**DOI:** 10.1080/0886022X.2022.2162417

**Published:** 2023-01-12

**Authors:** Zhen Li, Tao Zhen, Yong Zhao, Jianbin Zhang

**Affiliations:** a School of Public Health and Management, Chongqing Medical University, Chongqing, China; b Department of Nephrology, Yongchuan Hospital, Chongqing Medical University, Chongqing, China; c Department of Nephrology, The Affiliated Banan Hospital of Chongqing Medical University, Chongqing, China

**Keywords:** End-stage kidney disease, dialysis, nutrition literacy, reliability, validity, quality of life, malnutrition

## Abstract

**Objectives:**

To construct a valid and reliable Nutritional Literacy Scale for patients with end-stage kidney disease (ESKD) receiving dialysis and evaluate associations between nutrition literacy and quality of life.

**Methods:**

A total of 208 ESKD patients receiving dialysis were selected for this study. Nutrition literacy evaluation items were drafted based on dietary guidelines for chronic kidney disease (CKD), Literature reviews and expert consultation. Scale reliability and validity were then assessed. Factors influencing nutrition literacy and the associations among nutrition literacy, nutritional status, and quality of life were evaluated.

**Results:**

The scale consists of 28 items with a scale-level content validity index of 0.91 and item-level content validity indices ranging from 0.83 to 1.00. Factor analysis identified 4 common factors (dimensions) named nutrition knowledge, cognitive attitude, behavioral practice, and information acquisition ability that collectively explained 56.31% of literacy score variation. The overall Cronbach’s α coefficient of the scale was 0.83, the dimensional Cronbach’s α coefficients ranged from 0.79 to 0.87, and the retest reliability was *r* = 0.73 (*p* < 0.05). Age, education level, residence (urban vs. Rural) , occupational status and dialysis modalities were significant factors influencing nutrition literacy. Nutrition literacy score was negatively correlated with SGA score and positively correlated with serum albumin and prealbumin concen- trations, and with SF-36 quality of life score (all *p* < 0.05).

**Conclusions:**

This new Nutrition Literacy Scale demonstrates high reliability and validity for Chinese ESKD patients undergoing dialysis. The nutrition literacy is influenced by age, education level, residence, occupational status and dialysis modalities, associated not only with nutritional status but also with quality of life.

## Introduction

According to recent epidemiological surveys, the incidence of chronic kidney disease (CKD) has reached 15.1% in the United States and 10.8% in China, where it is the fourth most common chronic condition after hypertension, diabetes, and tumors [1,2]. Further, CKD is considered a global threat to public health. Since CKD is irreversible, many patients will ultimately reach end-stage kidney disease (ESKD) requiring kidney replacement therapy (KRT). Hemodialysis (HD) and peritoneal dialysis (PD) are the main KRT for patients with ESKD, and both require concomitant nutritional and diet therapy for optimal efficacy. Mastering the required nutritional knowledge and self-management skills can not only prevent nutritional deficiency but may also prolong the time window of effective dialysis treatment [2,3].

The nutritional management of diseases such as CKD requires nutritional literacy, which Blitstein and Evans defined as ‘the capability of individuals to obtain and understand nutrition facts panel (NFP) information, make accurate decisions, and use NFP information to maintain and promote the nutritional status of themselves and others’ [4]. Nutritional literacy will influence patients’ selection of dietary nutrients and dietary patterns, thereby influencing the progression of CKD [5,6]. Offering dietary guidance and improving nutritional literacy for CKD patients can prevent anemia, control electrolyte and acid-base disorders, reduce water-sodium retention, mitigate abnormal mineral and bone metabolism, and ultimately delay the need for dialysis [7]. However, a reliable evaluation tool is urgently required to measure the nutritional literacy of ESKD patients currently receiving dialysis.

Nutritional literacy is strongly correlated with the quality of life of patients with chronic diseases such as diabetes mellitus, hypertension, hyperlipidemia, and obesity [3]. Improving nutritional literacy can also enhance the treatment drug efficacy, effectively ease fatigue, anxiety, and depression, and elevate the quality of life of patients with chronic diseases [8,9]. In the present study, a new Nutritional Literacy Scale is described and validated. Using this scale, we then examine if nutritional literacy can improve the quality of life and nutritional status of ESKD patients receiving dialysis.

## Subjects and methods

### Patients

A total of 208 ESKD patients currently receiving maintenance HD (*n* = 156) or PD (*n* = 52) at two dialysis centers in Chongqing, China, from January 2019 to December 2021 were selected as candidates for this study. Inclusion criteria were as follows: (a) meeting diagnostic criteria for ESKD according to guidelines developed by the Kidney Disease Outcomes Quality Initiative (KDOQI) Working Group [10] and requiring dialysis treatment; (b) complete clinical data available, (c) clear consciousness, basic listening, speaking, reading, and writing skills, and able to read and understand the questionnaire content by themselves or with the help of others; (d) willing to provide informed consent and cooperate with the investigation. Exclusion criteria were (a) severe mental illness or cognitive impairment, (b) pregnant or lactating, and (c) Patients with tumor, severe infection, eating difficulty and other factors influencing nutritional status. The demographic characteristics of the selected patients are summarized in Table 1. This study was reviewed and approved by the Ethics Committee of Chongqing Medical University (No.2020(315)). Informed consent was exempted since the study only involved an analysis of anonymized existing data and records.

**Table 1.** Demographic characteristics of subject.

Items	Group	*N*	Frequency (%)
Age (years)	≤50	93	44.7
	51 ∼ 60	67	32.2
	≥61	48	23.1
Sex	Male	124	59.6
	Female	84	40.4
Education level	Primary and below	87	41.8
	Junior middle school	68	32.7
	Junior College	20	9.6
	College degree and above	33	15.9
Occupational status	Employed	27	13.0
	Unemployed	149	71.6
	Retired	32	15.4
Registered residence	Urban	92	44.2
	Rural	116	55.8
Marital status	Married	172	82.7
	Divorce	8	3.8
	Unmarried	14	6.7
	Widowed	14	6.7
Dialysis methods	hemodialysis	156	74.5
	Peripherol dialysis	52	25.5
Total		208	100.0

## Methods

### Creation of the initial item Pool

An initial pool of 32 items was compiled based on a health literacy level model [11], the theory of knowledge, behavior, and belief (KABP) [12], Clinical Practice Guidelines for Nutrition in CKD from the KDOQI [13], clinical practice guidelines on undernutrition in CKD [14], the Japanese Nutrition Literacy Scale [15], other literature reviews [2,5,16], and in-depth interviews with patients receiving maintenance dialysis. Two rounds of Delphi consultation were then conducted with 12 experts selected according to the following criteria: (a) engaged in CKD research for ≥3 years or nutrition research for ≥5 years and with a senior professional title and doctoral degree, and (b) able to provide comments on item modifications within the specified time period. According to expert feedback and further discussions among research group members, 4 items were deleted, yielding a preliminary 28-item nutritional literacy scale.

### Clinical investigation

#### Sample size estimation

According to the principle that the sample size should be greater than 5 times the number of variables, 208 patients were enrolled.

#### Investigation method

Scale assessors received uniform training and additional explanations were provided to insure that patients understood each item.

#### Pilot investigation

Thirty patients were randomly selected for the pilot investigation and language adaptation of the preliminary (32-item) questionnaire, and adjustments were made by considering the following issues:‘ Can the patients understand the contents of the item?’, ‘How do they interpret the content? Is the content of the scale ambiguous?’, and ‘Does it take too long?’ In addition, patients’ opinions on the scale were collected, and ambiguous items or passages were modified. Finally, the prediction scale was developed. The average duration for scale completion was about 12 min.

#### Formal investigation

**Figure 1.**
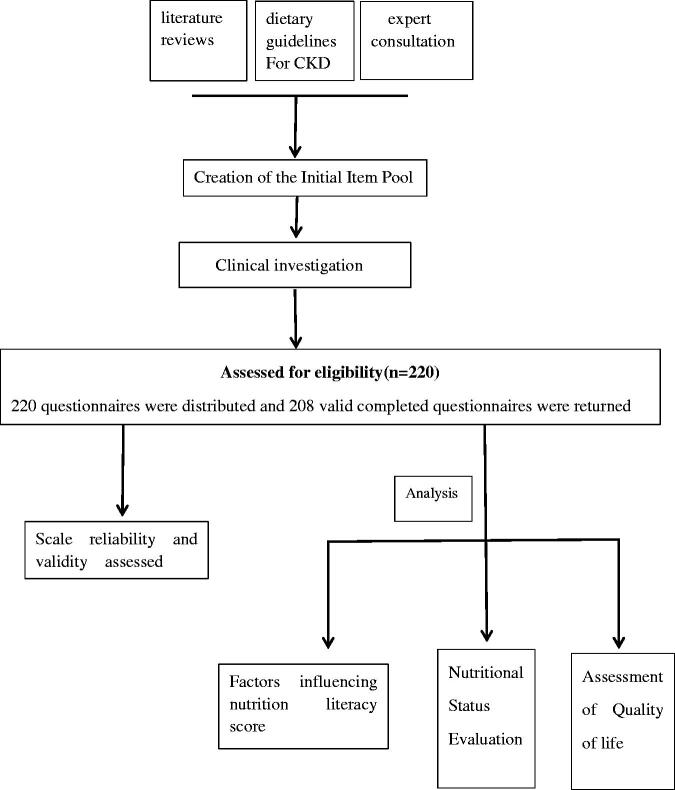
Flow chart Development and assessment of a nutrition literacy scale.

In total, 220 questionnaires were distributed and 208 valid completed questionnaires were returned, including 52 from PD patients and 156 from HD patients. The other twelve questionnaires were deemed invalid due to incomplete responses (Figure 1).

### Nutritional status evaluation

The Subjective Global Assessment (SGA) scale recommended by the U.S. National Kidney Foundation Kidney Disease/Dialysis Outcomes and Quality Initiative (KDOQI) is the most common tool used to diagnose malnutrition in patients with CKD and those undergoing maintenance dialysis [17]. In the current study, patients were divided into three groups according to overall SGA score: group A with SGA score ≤ 3 (well nourished), group B with SGA scores from 4 to 8 (moderate malnutrition), and group C with SGA score ≥9 (severe malnutrition). Serum albumin and prealbumin levels were also measured on SGA evaluation day.

### Assessment of quality of life

Quality of life was assessed using the original Chinese SF-36 scale of the International Quality of Life Assessment (IQOLA) Project introduced by professor Li Lu and colleagues. The Chinese version was developed by considered the unique aspects of Chinese culture, and a regional norm was established. The health concept represented by this scale is applicable to the healthy population and patients with chronic diseases [18]. Further, the SF-36 possesses high reliability, validity, and responsiveness to the quality of life of patients undergoing dialysis [19]. The 36 questions cover 8 dimensions of quality of life. For each dimension, scores range from 0 to 100, with a higher score indicating better quality of life.

### Quality control

Investigators conducted repeated pretesting before the survey. Investigators asked questions, provided pilot study subjects with explanations as needed, and performed data collection, review, coding, and entry according to preset standards.

### Statistics

A database of subject characteristics and questionnaire responses was established using Epidata 3.1, and all results were analyzed using SPSS 25.0 (International Business Machines Corporation, Chicaco, USA). Categorical variables are expressed as numbers or frequencies (%) and continuous variables as mean ± standard deviation (SD). Two group means were compared by independent samples t-test and multiple means by analysis of variance. Internal consistency reliability of the scale was evaluated by Cronbach’s α coefficient and external consistency by Pearson’s correlation between test and retest scores. A scale-level content validity index (S-CVI) and item level content validity indices (I-CVIs) were also calculated. In addition, exploratory factor analysis and Pearson’s correlation analysis between factors and total scores were used to examine structural validity. AP ≤ 0.05 was considered significant for all tests.

## Results

### Content validity

Two rounds of Delphi consultation were conducted with 12 subject experts, and recommended modifications were made to the original pool of items. According to expert opinion, the modified items more clearly reflect the nutritional literacy of patients and are easier to understand. Further, the items show good content validity at both the whole-scale level (S-CVI of 0.86) and at the individual item level CVIs ranging from 0.83 to 1.00.

### Construct validity

Exploratory factor analysis was conducted to ensure that the scale items also have good structural validity. First, Bartlett’s spherical test was used to confirm suitability for factor analysis (χ2 = 2031.51, *p* < 0.001, and KMO = 0.83). Common factors were then extracted by principal component analysis, and the maximum variance was used for rotation. Four factors were extracted according to eigenvalues > 1. Four items with load values lower than 0.50 and close within a dimension were eliminated, and the newly refined 28-item scale was re-analyzed. Again, 4 common factors were extracted that accounted for 56.31% of score variation among participants (see Table 2).

**Table 2.** The composition of items in the Nutritional Literacy Evaluation Scale for ESKD patients on dialysis and the results of factorial analysis.

	Factors			
Items	1	2	3	4
K1. Limit the intake of plant protein and supplement highquality protein (such as fish, milk, eggs, etc.)	0.872			
K2. Use wheat starch (or other starches) as the staple food to partially replace ordinary rice or flour	0.765			
K3. Calculate the energy intake based on your own activity level and weight	0.864			
K4. Appropriately increase the intake of carbohydrates on the basis of reasonable energy intake (People with abnormal glucose metabolism should limit the intake of refined sugar)	0.752			
K5. Avoid high-protein diets for patients with chronic kidney disease	0.837			
K6. Eat vegetable oils instead of animal fats	0.659			
K7. Limit high-phosphorus foods and carefully select animal liver, nuts, dried beans, and processed foods containing phosphorus	0.726			
K8. Limit foods high in potassium and carefully choose oranges, bananas, mangoes, et	0.568			
K9. Low-salt diet (or daily salt intake of dialysis patients	0.791			
K10. Dialysis patients should be supplemented with natural vitamin D and common vitamin D-rich foods	0.673			
K11. Appropriately limit water intake to maintain a balance between intake and output	0.826			
K12. Daily intake of dietary fiber	0.652			
A1. Do you think diet management is very important in CKD		0.783		
A2 Do you think that dietary adjustment in patients with CKD is helpful to delay the deterioration of renal function		0.695		
A3. Are you willing to change your eating habits because of your illness		0.654		
A4. Do you think people with CKD should avoid high-protein diets		0.598		
A5. Is it necessary to limit the intake of water and sodium for patients with CKD		0.671		
B1. Will you take the initiative to learn nutrition knowledge related to CKD			0.826	
B2. Do you choose the right diet every day, or special treatment of the diet (removal of excess water, potassium, phosphorus, etc.)			0.815	
B3. Do you take the time and effort to acquire or consult about nutrition			0.739	
B4. Do you pay attention to the nutrition label when you buy food			0.802	
B5. When you cook whether can achieve less oil cooking			0.782	
B6. Do you use wheat starch (or other starch) as the staple food, to partially replace the ordinary rice or flour			0.727	
I1. I know where to look for information on nutrition				0.854
I2. I can understand the nutritional information I am exposed to in my daily life				0.841
I3. I often discuss nutrition-related knowledge with patients or family members				0.799
I4. I will discuss dietary issues with professionals (nutrition experts, community doctors, nurses, etc.)				0.701
I5. Some nutritional problems that I don’t understand will continue to be asked				0.801
Explained Variations (%)	23.294	12.647	11.281	9.087
Cumulative contribution (%)	23.294	35.941	47.222	56.309

Based on discussions among research group members and considering the health literacy level model [8] and KABP [20], the four common factors (dimensions) were named ‘nutrition knowledge level’, ‘cognitive attitude’, ‘behavioral practice’, and ‘information acquisition ability’. The final questionnaire includes two parts, basic information and nutritional literacy. Basic information gathered includes sex, age, marital status, occupational status, household registration (urban vs. rural), education level, and dialysis time. The nutritional literacy component includes 12 items on nutrition knowledge level (items 1–12), 5 items on cognitive attitude (items 13–17), 6 items on behavioral practice (items 18–23), and 5 items on information acquisition ability (items 24–28). Each correct item response is worth 1 point, for a total score of 28. A total score ≤ 16 is considered indicative of low nutritional literacy, 16 to 25 of average nutritional literacy, and ≥ 25 of high nutritional literacy.

### Internal consistency reliability

Cronbach’s α coefficient is used to describe the internal consistency of a scale, with a value > 0.8 generally considered acceptable [14]. Table 3 shows the Cronbach’s α coefficients for all items grouped according to the dimensions defined by factor analysis. Both the total Cronbach’s α for the 28 items (0.83), and the Cronbach’s α values for most individual items exceed 0.8 (range 0.79–0.87). Thirty subjects were retested after an interval of 2 weeks, and the test–retest reliability estimated by Pearson’s correlation coefficient. The test-retest reliability coefficient of the scale is 0.73, and the test-retest reliability coefficient of each factor ranges from 0.68 to 0.82 (Table 4), which is considered acceptable [21].

**Table 3.** Internal consistency of the scale.

Dimensions	Items	Cronbach’s α
Nutrition knowledge level	12	0.814
Cognitive and attitude	5	0.791
Behavior practice ability	6	0.865
Information acquisition ability	5	0.802
Total table	28	0.827

**Table 4.** Test-retest reliability of the scale.

Dimensions	items	r	*p* Value
Nutrition knowledge level	12	0.821*	0.001
Cognitive and attitude	5	0.760*	0.013
Behavior practice ability	6	0.741*	0.041
Information acquisition ability	5	0.682*	0.020
Total	28	0.753*	0.016

*p < 0.05.

### Associations of nutritional literacy scores with demographic characteristics

The average Nutritional Literacy Score of the entire patient cohort was 18.8 ± 4.1 points, with most individual scores in the low to medium range (16–25). Scores did not differ by sex or marital status (*p* > 0.05) but did differ by age group, household registration (urban vs. rural), education level, employment status and dialysis methods (*p* < 0.05). There was also a significant negative correlation between patient age and nutritional literacy score. As expected, higher education level was associated with greater Nutritional Literacy Score. Collectively, younger, working, educated urban patients demonstrated generally higher scores than unemployed, older, rural-dwelling patients with less education (Table 5).

**Table 5.** Demographic characteristics and nutritional literacy scores (*n*, ′x ± s).

Items	Group	*N*	Frequency (%)	Nutritional literacy score (mean ± SD)	t/F	*p* Value
Age (years)					6.04	0.030
	≤50	93	44.7	19.8 ± 4.0		
	51 ∼ 60	67	32.2	18.1 ± 3.8		
	≥61	48	23.1	17.6 ± 4.4		
Sex					−1.56	0.113
	Male		59.6	18.4 ± 4.3		
	Female		40.4	19.3 ± 3.9		
Education level					4.97	0.020
	Primary and below	87	41.8	17.6 ± 4.1		
	Junior middle school	68	32.7	19.0 ± 3.7		
	Junior College	20	9.6	20.1 ± 3.4		
	College degree and above	33	15.9	20.4 ± 4.6		
Occupational status					5.09	0.007
	Employed	27	13.0	20.7 ± 3.7		
	Unemployed	149	71.6	18.2 ± 4.0		
	Retired	32	15.4	19.6 ± 4.4		
Registered residence					3.19	0.001
	Urban	92	44.2	19.8 ± 3.9		
	Rural	116	55.8	18.0 ± 4.1		
Marital status					0.25	0.861
	Married	172	82.7	18.8 ± 4.2		
	Divorce	8	3.8	19.5 ± 3.7		
	Unmarried	14	6.7	18.8 ± 3.8		
	Widowed	14	6.7	18.8 ± 4.1		
Dialysis methods					−4.12	0.000
	hemodialysis	155	74.5	18.1 ± 4.0		
	Peripherol dialysis	53	25.5	20.7 ± 3.8		

### Correlation between nutritional literacy score and nutritional assessments

Total Nutritional Literacy score was significantly and negatively correlated with SGA score, implying that lower nutritional literacy increases the likelihood of malnutrition. In accord with this result, nutritional literacy was significantly and positively correlated with serum albumin and serum prealbumin concentrations (all *p* < 0.05) (Table 6). The SGA score was highest in the low nutritional literacy group (score > 16) followed by the intermediate and high nutritional literacy groups, and the differences were statistically significant. Based on SGA results, only 2 patients met the diagnostic criteria for severe malnutrition (group C). Hence, these subjects were included with SGA group B to form a malnutrition group while the remaining subjects (group A) were included in the well-nourished group. The incidence of malnutrition was significantly lower in the high nutritional literacy group compared to the intermediate and low nutritional literacy groups (11.6% vs. 28.0% and 63.8%, respectively) and also significantly lower in the intermediate group than the low literacy group (Table 7).

**Table 6.** The correlation of nutritional literacy score with SGA、serum concentrations of albumin and prealbumin.

nutritional assessments	correlation	*p* Value
the Subjective Global Assessment	−0.872	0.028
Serum albumin (g/L)	0.674	0.036
Serum prealbumin (mg/L)	0.741	0.020

**Table 7.** Correlation between different nutritional literacy levels and nutritional status.

nutritional assessments	Low nutritional literacy group (<16) *N* = 47	Medium nutritional literacy group (16-25) *N* = 118	High nutritional literacy group (>25) *N* = 43	F/χ２	*p* Value
the Subjective Global Assessment (x ± s)	6.64 ± 5.01	4.63 ± 3.70	2.48 ± 2.27	6.87	0.032
Good nutrition *n*(%)	17 (36.2%)	85 (72.0%)	38 (88.4%)	22.56	0.011
malnutrition *n*(%)	30 (63.8%)	33 (28.0%)	5 (11.6%)		

### Correlation between nutritional literacy score and quality of life

**Figure 2.**
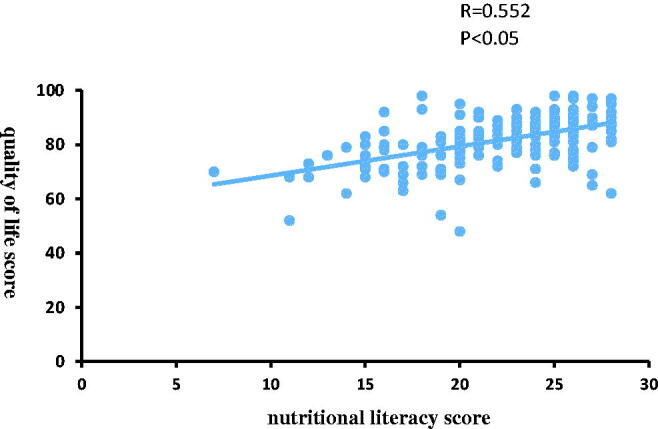
Association between nutritional literacy score and quality of life score.

The SF-36 quality of life scores were poorest in the low nutritional literacy group, significantly improved in the intermediate group, and greatest in the high nutritional literacy group (Table 8). Moreover, correlation analysis revealed a strongly significant association between Nutritional Literacy Score and quality of life score (*R* = 0.55, *p* < 0.01) (Figure 2).

**Table 8.** Correlation between nutritional literacy level and quality of life.

	Low nutritional literacy group (<16) *N* = 47	Medium nutrition literacy group (16–25) *N* = 118	High nutritional literacy group (>25) *N* = 43	F/χ２	*p* Value
Assessment of Quality of life	75.0 ± 11.8	83.6 ± 13.5	89.3 ± 13.6	7.62	0.033

## Discussion

Nutritional literacy promotion has the potential to reduce the incidence and improve the self-management of diabetes, CKD, hypertension, overweight, and obesity, decrease the risk of breast cancer, and slow functional decline during aging [3,10,20,22–25]. However, current nutritional literacy evaluation tools such as Nutritional Literacy Scale (NLS) [10], Nutrition Literacy Assessment Instrument (NLAI) [26], and Japanese Nutritional Literacy Scale [27] may not be optimal for the evaluation of ESKD patients on dialysis.

The Nutritional Literacy Evaluation Scale for ESKD patients described in this study is based on the health literacy level model and the theory of KABP [28], with content derived mainly from nutritional dietary guidelines for CKD, related literature reviews [13,14,16], and expert opinion. The final scale covers four aspects (domains) of nutritional literacy: nutritional knowledge level (knowledge), cognitive attitude (attitude), behavioral practice (behavior), and information acquisition ability (interactivity). The S-CVI/AVE (0.91) and all I-CVIs (0.83–1.00) were above the thresholds for good content validity according to a previous questionnaire development study [21]. The Cronbach’s α coefficient of the total scale was greater than 0.80, and the Cronbach’s α coefficient of each dimension ranged from 0.79 − 0.87, indicating good internal consistency. Further, scale retest reliability was high (*r* = 0.75, *p* < 0.05) after 2 weeks. Finally, literacy scores were positively correlated with serum albumin and prealbumin concentrations, which are common clinical measures of nutritional status. Serum albumin level in a strong predictor of prognosis and premature mortality among dialysis [29]. Therefore, nutritional literacy level as measured by this new test appears to reflect dietary preference, dietary patterns, and the nutritional status of these patients.

Nutritional knowledge subscores indicated that the vast majority of patients were unaware of appropriate energy intake levels, the use of wheat starch to replace other forms of starch, and the benefits of vitamin D supplementation for CKD. In addition, most patients do not consult nutritional knowledge resources or pay attention to nutrition labels on food packaging as indicated by poor performance on behavioral practice and information acquisition ability items. There was also no positive correlation between cognitive attitude score and behavioral practice score, indicating that cognitive attitude was not the only factor influencing patient dietary behavior. Indeed, there were statistically significant differences in literacy scores among subgroups stratified by age, household registration (urban vs. rural), dialysis mode, education level, and employment status (but not sex, dialysis time, or marital status). Total score and all dimension scores were negatively correlated with age, indicating that older patients generally have lower nutritional literacy. Conversely, literacy was positively correlated with educational level. However, age was also negatively correlated with education level, so poor literacy among the aged likely reflects in part fewer educational opportunities in previous generations. In addition, the high scores among urban patients compared to rural patients and of the employed compared to the unemployed likely also reflect educational attainment as 85% of the employed patients had a junior high school education or above. Unexpectedly, Nutritional Literacy Scores also differed among dialysis modality subgroups, with PD patients demonstrating greater literacy than HD patients, possibly due to PD patients experienced more personal control and had a better understanding of the illness, self-management of patients on peritoneal dialysis develops a sense of personal control which correlates positively and significantly with many aspects of quality of life [30].

Malnutrition is a frequent complication of ESKD. Subjective assessments of nutrition including subjective global assessment (SGA) and malnutrition-inflamm- ation score (MIS) have been proved to be some of the strongest predictors of clinical outcomes in the dialysis population [31]. Our study revealed that SGA score was significantly lower and serum albumin and prealbumin concentrations significantly greater in the high nutritional literacy group than the low nutritional literacy group, suggesting that poor nutritional literacy can result in malnutrition of CKD patients receiving dialysis. It has been suggested that nutritional literacy promotes improved dietary quality and habits. Intake of specific dietary nutrients and appropriate dietary patterns strongly influence the progression and treatment of chronic diseases [29]. The current study suggests that enhancing the nutritional literacy of CKD patients can prevent inappropriate dietary habits resulting in malnutrition, potentially reducing the risks of complications.

In the present study, the Chinese SF-36 scale was adopted to assess ESKD patient quality of life as this tool has demonstrated good reliability and validity for healthy and chronic disease groups, including Chinese patients undergoing dialysis [19]. The level of nutritional literacy was positively correlated with quality of life score, indicating that nutritional literacy can improve the efficacy of dialysis treatment, while dialysis patients with low nutritional literacy likely have poor self-management capabilities, increasing malnutrition risk and limiting therapeutic efficacy. Specifically, insufficient intake of high-quality proteins and excessive intake of phosphorus, sodium, potassium, lipids, and water may increase the incidence of complications, thereby reducing quality of life and possibly also long-term survival [32].

This study has several limitations. The sample size is relatively small, so results may be influenced by selection bias. In addition, many factors influencing nutritional literacy may have been missed. As stated, this test must be examined and validated in multiple cohorts with different demographic and clinical characteristics. Scales responses are also subjective, although scores are strongly correlated with objective measures of nutritional status like serum albumin. In the next step, a multicenter clinical study will be conducted to provide a more solid theoretical basis for use of this Nutritional Literacy Scale in clinical practice.

## Conclusion

We have developed a Chinese Nutritional Literacy Scale for end-stage kidney disease patients receiving dialysis with good overall reliability and validity. The reliability and validity of this scale must be verified in additional study cohorts and in clinical practice. This foundational study suggests that the overall nutritional literacy of Chinese ESKD patients receiving dialysis is low, especially older rural patients with limited formal education. The Nutritional Literacy Scale associated not only with nutritional status but also with quality of life.

## Data Availability

The data used to support the findings of this study are restricted by the Ethics Committee of School of Public Health and Management, Chongqing Medical University to protect patient privacy. Data are available from School of Public Health and Management, Chongqing Medical University for researchers who meet the criteria for access to the data.
